# MKP1 mediates chemosensitizer effects of E1a in response to cisplatin in non-small cell lung carcinoma cells

**DOI:** 10.18632/oncotarget.6574

**Published:** 2015-12-12

**Authors:** Francisco J. Cimas, Juan L. Callejas-Valera, Raquel Pascual-Serra, Jesus García-Cano, Elena Garcia-Gil, Miguel De la Cruz-Morcillo, Marta Ortega-Muelas, Leticia Serrano-Oviedo, J. Silvio Gutkind, Ricardo Sánchez-Prieto

**Affiliations:** ^1^ Unidad de Medicina Molecular, laboratorio de Oncología, Centro Regional de Investigaciones Biomédicas, Universidad de Castilla-La Mancha, 02006, Albacete, Spain Unidad de Biomedicina UCLM-CSIC; ^2^ Moores Cancer Center/UCSD, La Jolla, CA 92093-0819, USA

**Keywords:** E1a, MKP1, cisplatin, chemotherapy, lung cancer

## Abstract

The adenoviral gene E1a is known to enhance the antitumor effect of cisplatin, one of the cornerstones of the current cancer chemotherapy. Here we study the molecular basis of E1a mediated sensitivity to cisplatin in an experimental model of Non-small cell lung cancer. Our data show how E1a blocks the induction of autophagy triggered by cisplatin and promotes the apoptotic response in resistant cells. Interestingly, at the molecular level, we present evidences showing how the phosphatase MKP1 is a major determinant of cisplatin sensitivity and its upregulation is strictly required for the induction of chemosensitivity mediated by E1a. Indeed, E1a is almost unable to promote sensitivity in H460, in which the high expression of MKP1 remains unaffected by E1a. However, in resistant cell as H1299, H23 or H661, which display low levels of MKP1, E1a expression promotes a dramatic increase in the amount of MKP1 correlating with cisplatin sensitivity. Furthermore, effective knock down of MKP1 in H1299 E1a expressing cells restores resistance to a similar extent than parental cells.

In summary, the present work reinforce the critical role of MKP1 in the cellular response to cisplatin highlighting the importance of this phosphatase in future gene therapy approach based on E1a gene.

## INTRODUCTION

The adenoviral protein E1a was primary described as an oncogene able to induce transformation in cooperation with other oncogenes such as Ras or Myc [1]. However, E1a also has an antitumor effect exerted by different mechanism as reversing the transformed phenotype, inhibiting metastasis, or inducing apoptosis in different experimental models [2–4] (for a review see [5]). From the therapeutic point of view is well stablished that E1a is able to promote radio/chemosensitivity [6–9]. In this regard, the ability of the adenoviral protein to induce sensitivity could be explained by different mechanisms. For example, the loss of function in the PI3K/AKT pathway has been proposed to be a key step in the induction of chemo/radiosensitivity [10, 11]. It is also known that this adenoviral protein is able to induce p53 stabilization [12] through binding to Mdm4, rendering Mdm2 inhibition and decreasing nuclear export and degradation of p53 [13]. However, the chemosensitizer ability of E1a also has been reported to be p53 independent [14]. On the other hand, the effect exerted onto other critical tumor suppressors genes has been also proposed to be involved in E1a-induced chemosensitization, such p19ARF [12] or pRb. [15] In addition, proapoptotic protein as Bax and Caspase-9 [16–18] that ordinarily promotes cell death, could account for E1a associated sensitivity. It is also noteworthy that some of the biological properties of E1a are related to mitogen-activated protein kinases (MAPKs) signaling pathways. E1a is known to interfere with the p38 pathway in response to different stimuli such as UV, chemo and radiotherapy playing an important role the inhibition of the PI3K/AKT pathway through the protein phosphatase PP2A [11, 19]. In addition, E1a is able to block ERK1/2 activation in fibroblast in the presence of v-H-Ras by increasing the levels of MKP1, a nuclear phosphatase for MAPK, explaining the ability of E1a to escape from Ras induced senescence [20]. Finally, E1a is also know to affect JNK in a Rac upstream manner but no phosphatase has been implicated in this case [21]. Nonetheless, all the previous suggest the important role that MAPKs and protein phosphatases could play in many of the biological properties controlled by E1a.

Non-small cell lung carcinoma (NSCLC) is a subtype of lung cancer, with a high ratio of refractory patients to the current therapy in which platin compounds are one of the cornerstones (for a review see [22]). Nonetheless, new approaches have been proposed to overcome cisplatin (cDDP) resistance, such as the use of glytazonas or copper chelators [23–25], aimed to improve platin based therapy, allowing a more selective use of the drug and avoiding some of the side effects. Interestingly an increase in the activity of the MAPKs has been linked with a more malignant phenotype. [26, 27]. In this regard, expression of MKP1, which is a key element to control the status of MAPKs pathways [28], has been shown to correlate with an improved survival for lung cancer [29].

In this scenario, we decided to study how E1a could promote chemosensitivity to cDDP in a panel of four NSCLC cell lines. Our results show how E1a, through the modulation of MKP1, promotes sensitivity in NSCLC derived cell lines, indicating that evaluation of MKP1 could be a key element for future E1a gene-therapy protocols in order to exploit the chemosensitizer properties of this gene.

## RESULTS

### E1a enhances the antitumor effect of cDDP in NSCLC trough promotion of apoptosis

**Figure 1:**
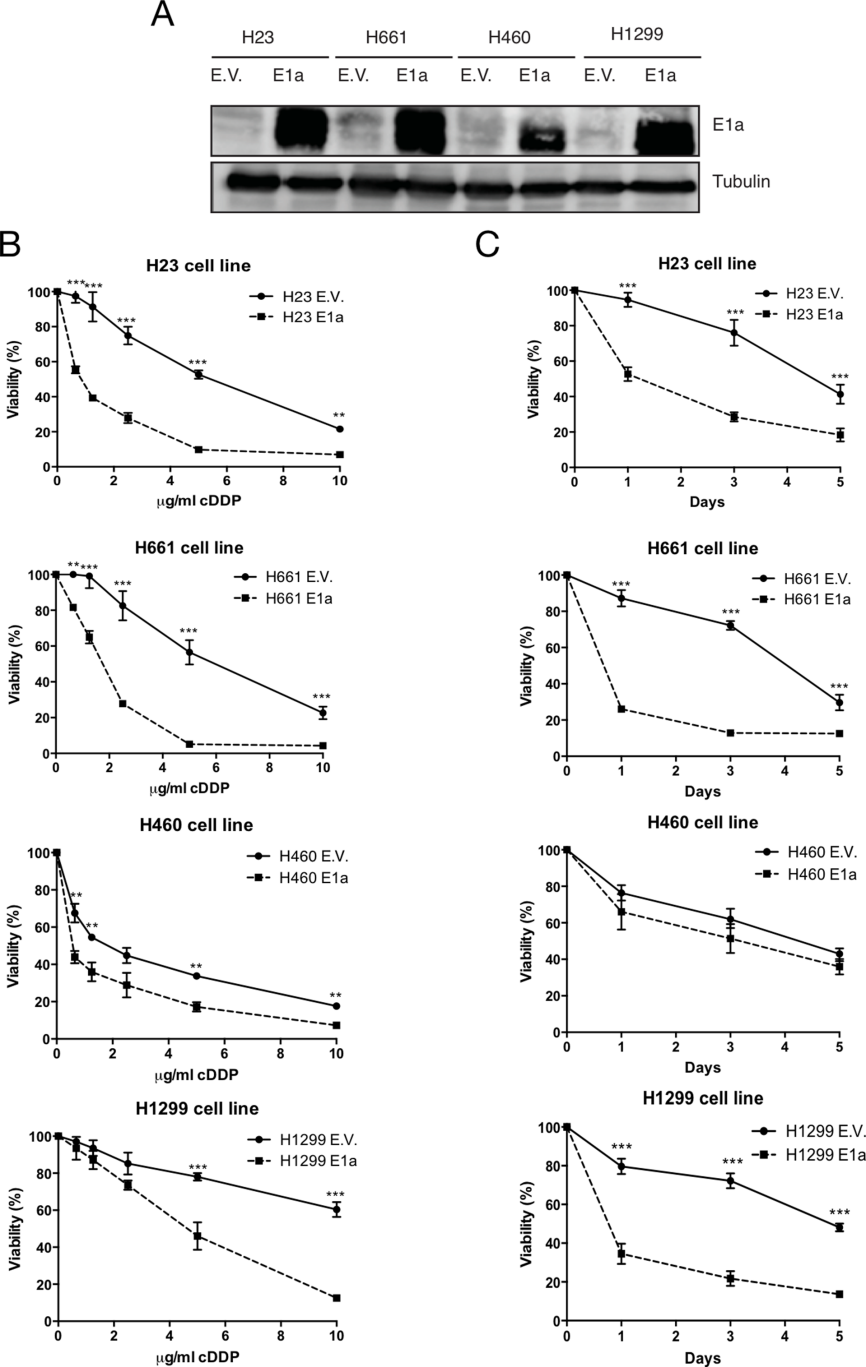
E1a promotes sensitivity to cDDP in NSCLC (**A**) H23, H1299, H460 and H661 were infected with empty vector or E1a 13s. 50 μg of total cell lysates (TCL) were blotted against E1a. Membranes were reproved against Tubulin as loading control. (**B**) NSCLC cell lines with/without E1a 13s were treated with the indicated doses of cDDP during 48 hours and viability was evaluated by crystal violet method. Bars indicate standard deviation (SD). (**C**) NSCLC cells lines carrying empty vector or E1a 13s were treated with the IC_75_ obtained from Figure 1B during 5 days and viability was evaluated by crystal violet method. Bars indicate standard deviation (SD).

In order to study the E1a mediated sensitivity to cDDP we decided to use an experimental model comprised of 4 NSCLC derived cell lines with different genetic backgrounds (H460, H23, H661 and H1299) [30]. Cells were infected with lentivirus carrying the adenoviral gene E1a (isoform 13s) and resistance to cDDP was evaluated. After achieving a successful expression of E1a in our cell lines (Figure 1A), cells were exposed to the indicated doses of cDDP during 48 hours. E1a was able to increase the sensitivity to cDDP in all cell lines, being maximum in H1299 cells. However, H460 cells showed a minimum effect as judged by crystal violet (Figure 1B) and confirmed by MTT assay (Supplementry Figure S1). Furthermore, the induction of chemosensitivity was also evaluated for the E1a 12s isoform, promoting again a chemosensitivity phenotype even in the most resistant model, such as H1299 cells (Supplementry Figure S2).

To normalize the cytotoxic effect of cDDP in our panel of NSCLC we calculated the specific IC_75_, according to the data obtained at 48 hours, for each parental cell line in response to cDDP. Then we performed time course assays from 24 up to 120 hours (Figure 1C). As expected, we found that sensitivity associated to E1a was clear in H1299, H23 and H661 compared with H460, in which E1a associated sensitivity to cDDP was again almost marginal.

**Figure 2:**
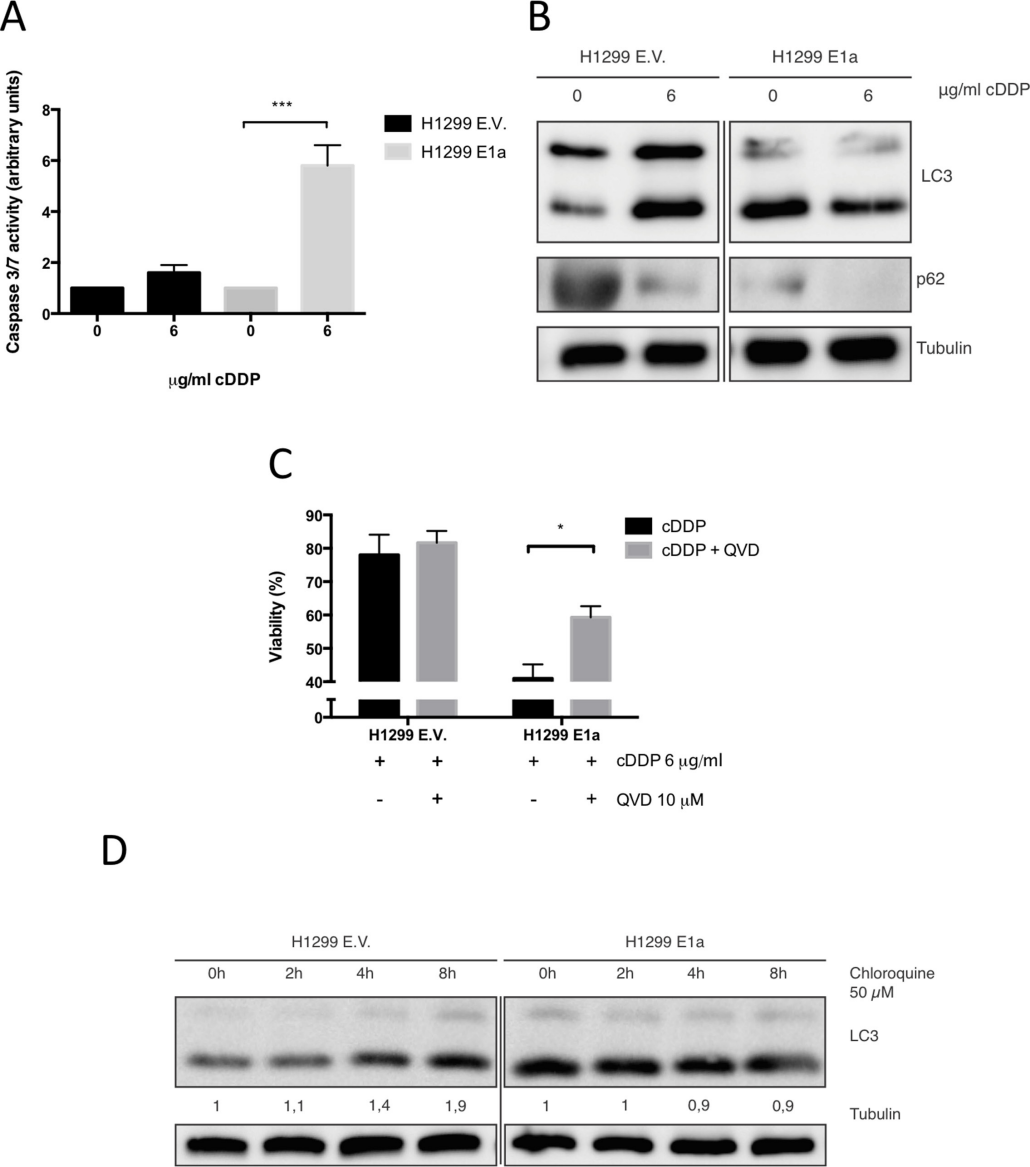
E1a enhances the antitumor effect of cDDP in NSCLC trough promotion of apoptosis (**A**) H1299 cells infected with E.V or E1a 13s were treated with 6 μg/ml of cDDP for 24 h and caspase 3/7 activity was evaluated. (**B**) H1299 cells infected with E.V or E1a 13s were treated with 6 μg/ml of cDDP for 48 h. Then, 50 μg of TCL were blotted against LC3 and p62. Membranes were reproved against tubulin as loading control. (**C**) Cells were treated as in (A) in the presence or absence of 10 μM Q-VD and then survival ratio was evaluated 48 hours later by using MTT assay. (**D**) H1299 cells infected with E.V. or E1a 13s were treated with chloroquine, in the absence of serum, at the indicated time points (time 0 means no chloroquine). Then 50 μg of TCL were blotted against LC3. Membranes were reproved against Tubulin as loading control.

As a result, we decide to study how E1a was affecting cell death mechanism triggered by cDDP in H1299 cells. As it is shown, (Figure 2A and 2B) E1a was able to promote an apoptotic response and blocks the induction of the characteristic autophagic response of resistant cells. Indeed, the use of QVD, a pan-caspase inhibitor [31], promotes a marked increase in the resistance of E1a expressing cells, but almost did not modify the response of the control cells (as it Figure 2C). Finally, autophagic flux was evaluated by using chloroquine [32]. As shown in Figure 2D, while in E1a expressing cells LC3 lipidation remains almost unaffected by the presence of chloroquine, non-expressing counterparts showed a marked increase in the lipidation of LC3, being consistent with a deregulation of the autophagic flux by the presence of E1a.

In summary, this set of data indicates that E1a is able to promote sensitivity by enhancing an apoptotic response to cDDP and blocking the characteristic induction of autophagy in our experimental model of H1299 cells.

### Sensitivity to cDDP correlates with MKP1 expression in NSCLC

**Figure 3:**
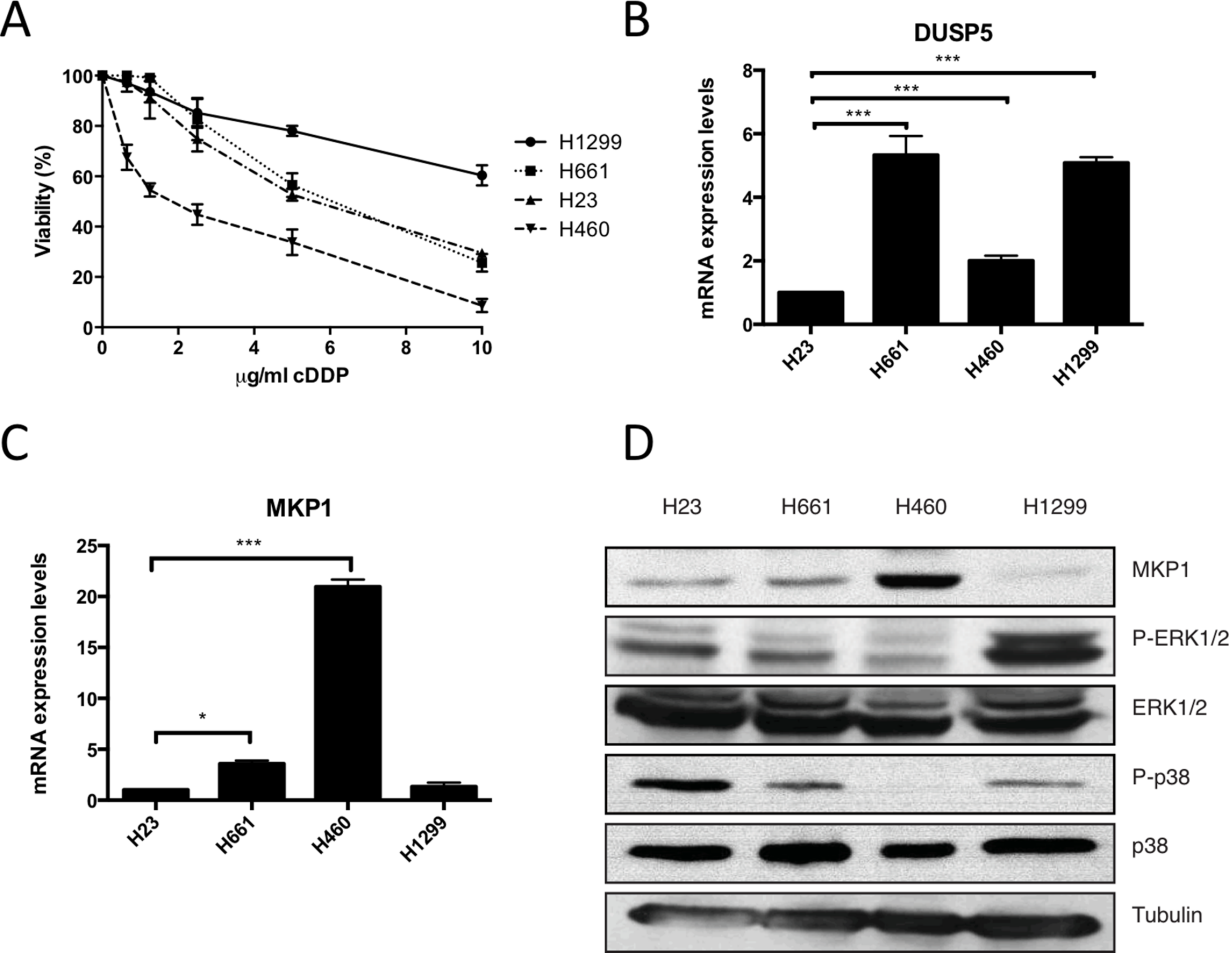
Sensitivity to cDDP correlates with low expression of MKP1 in NSCLC (**A**) NSCLC derived cell lines were treated with the indicated doses of cDDP during 48 hours and viability was evaluated by crystal violet method. Bars indicate standard deviation (SD). (**B**) H1299, H23, H661 and H460 were subjected to qRT-PCR assay in order to quantify DUSP5 mRNA expression levels. (**C**) Same approach was performed to measure MKP1 mRNA expression levels. (**D**) 50 μg of TCL from our panel of NSCLC cells were blotted against endogenous MKP1, p38MAPK and the respective active form. Tubulin was used as loading control.

Cellular response to cDDP has been connected with MAPKs signaling pathway (for a review see [33]). Among the several mechanisms to control MAPKs activity, protein phosphatases as MKP1 and DUSP5 are known targets of E1a [20]. Therefore, we decided to study the role of protein phosphatases in the chemosensitizer effect exerted by E1a in response to cDDP. Initially, we correlated the response of parental cell lines to cDDP (Figure 3A) with the expression levels by qRT-PCR of DUSP5 and MKP1 (Figure 3B and 3C). As it is shown, different levels of intrinsic resistance showed a nice correlation with MKP1, but not with DUSP5. Next we validated this data by checking protein levels of MKP1 in our panel of NSCLC derived cell lines, showing again a nice correlation (Figure 3D). Furthermore, although we failed to detect JNK phosphorylation (data not shown), we found that the expression level of MKP1 nicely correlated with p38 and ERK1/2 pathways activation in basal conditions, indicating the functionality of MKP1 expression [34].

**Figure 4:**
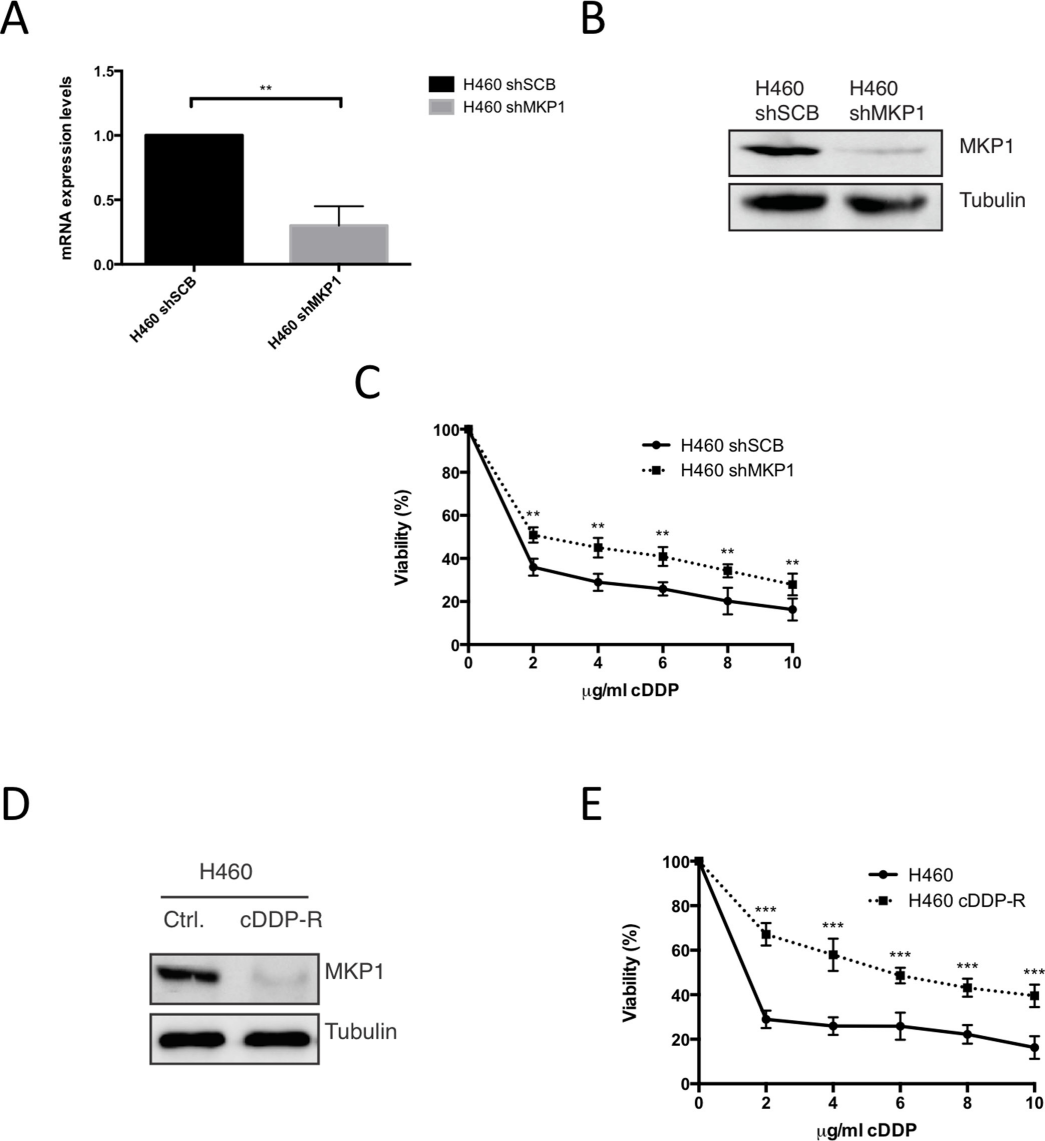
Abrogation of MKP1 mediates resistance to cDDP in NSCLC (**A**) Expression of MKP1 was evaluated in H460 infected with sh-RNA scrambled or MKP1 by qRT-PCR. (**B**) 50 μg of TCL from H460 cells infected with MKP1 sh-RNA or empty vector were blotted against MKP1. Tubulin was used as loading control. (**C**) H460 cell line infected with sh-RNA scrambled or MKP1 was treated with the indicated doses of cDDP during 48 hours and viability was evaluated by the crystal violet method. Bars indicate standard deviation (SD). (**D**) 50 μg of TCL from cDDP resistant H460 generated by co-culturing (cDDP-R) or parental H460 cell line were blotted against MKP1. Tubulin was used as loading control. (**E**) H460 cDDP-R or parental H460 were treated with the indicated doses of cDDP during 48 hours and viability was evaluated by crystal violet method. Bars indicate standard deviation (SD).

To fully probe the role of MKP1 in the cellular response to cDDP in our NSCLC derived cell lines model, we decided to knockdown MKP1 in H460 cell line. After achieve an effective knock down at the mRNA and protein level (Figure 4A and 4B), we found that MKP1 abrogation induce resistance to cDDP (Figure 4C), supporting the key role of MKP1 in cDDP sensitivity. Furthermore, we also performed an alternative approach generating a H460 resistant cell line by continuous exposure to cDDP. Interestingly this new cell line showed a marked decrease in the levels of this phosphatase that correlates again with resistance (Figure 4D and 4E).

Therefore, all these evidences support a critical role for MKP1 in the cellular response to cDDP, demonstrating how high levels of MKP1 correlate with sensitivity, while its suppression promotes resistance and suggesting that could be a potential mechanism to explain E1a associated chemosensitivity.

### Upregulation of MKP1 mediates E1a associated sensitivity to cDDP

**Figure 5:**
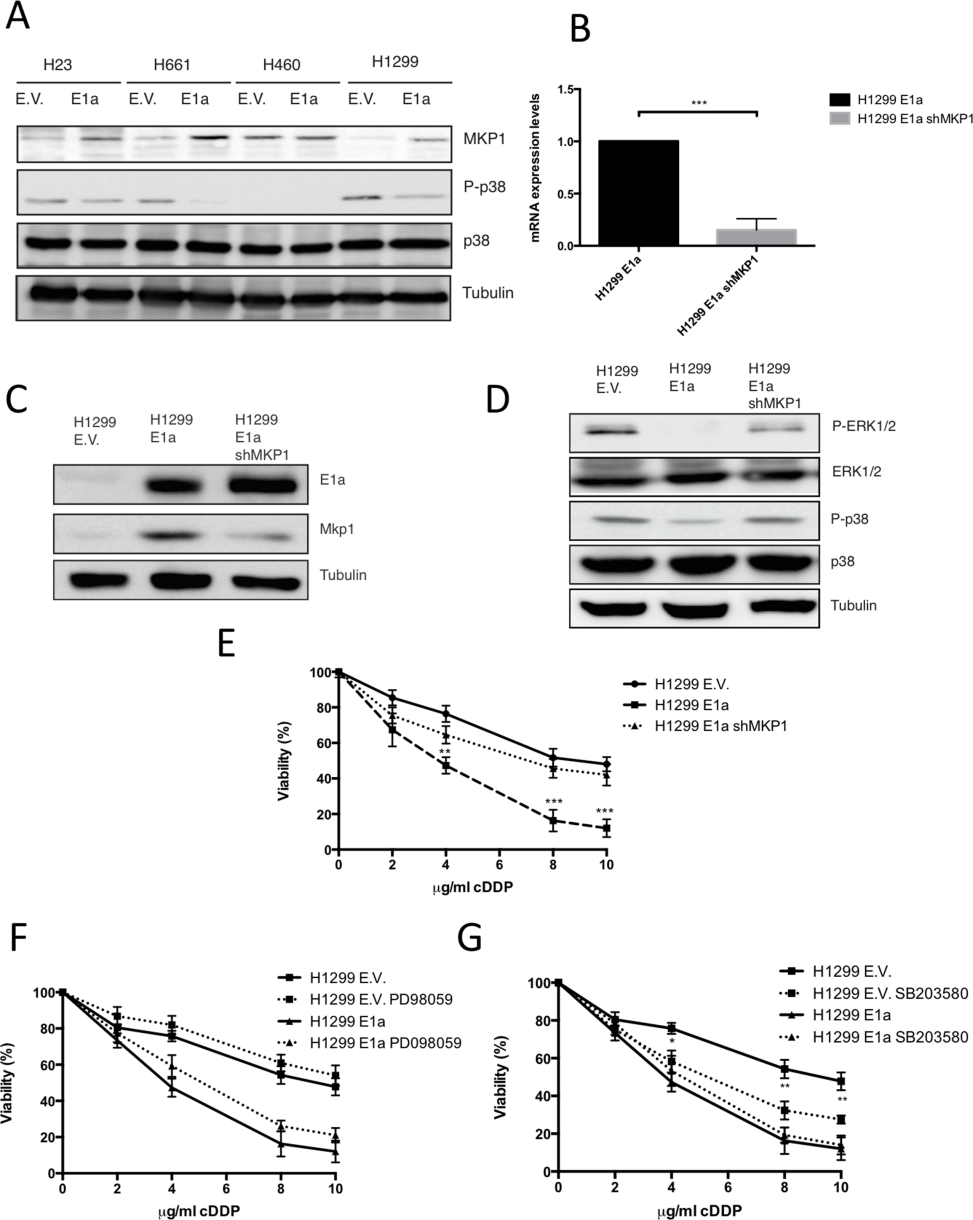
Upregulation of MKP1 mediates E1a associated sensitivity to cDDP in a p38MAPK dependent fashion (**A**) 50 μg of the TCL from NSCLC expressing E1a 13s or empty vector were used to evaluate the MKP1, P-p38MAPK and p38MAPK. Tubulin was used as loading control. (**B**) H1299 stably transfected with pcDNA E1a 13s (H1299 E1a) or empty vector (E.V.) were infected with lentivirus carrying sh-RNA against MKP1 (H1299 E1a shMKP1) and then mRNA MKP1 levels were evaluated by qRT-PCR. (**C**) 50 μg of TCL from cells used in B) were blotted against MKP1 and E1a. Tubulin was used as loading control. (**D**) 50 μg of TCL from cell used in C) were blotted against p38, ERK1/2 and the respective active forms. Tubulin was used as loading control. (**E**) H1299 E.V, H1299 E1a and H1299 E1a and sh-RNA MKP1 were treated with the indicated doses of cDDP during 48 hours and viability was evaluated by crystal violet method. Bars indicate standard deviation (SD). (**F**) H1299 E.V and H1299 E1a were treated with the indicated doses of cDDP during 48 hours in the presence/absences of PD98059 (10 μM). Viability was evaluated by crystal violet method. Bars indicate standard deviation (SD). (**G**) H1299 E.V and H1299 E1a were treated with the indicated doses of cDDP during 48 hours in the presence/absences of SB203580 (10 μM). Viability was evaluated by crystal violet method. Bars indicate standard deviation (SD).

Next, we tested whether MKP1 protein levels, in our model of NSCLC, were affected by E1a gene. As shown in Figure 5A, all the resistant cell lines showed a clear increase in the levels of MKP1 in the presence of E1a, except for H460 cells in which the presence of E1a did not modify the expression level of MKP1. Functionality of MKP1 alteration was evaluated by means of p38MAPK phosphorylation, showing a marked decrease except in H460 cells (Figure 5A).

To fully support our observations, we decided to abrogate MKP1 function in H1299 E1a expressing cells. Initially we tried some pharmacological approaches based on Ro 31–8220 [35] but renders an unacceptable toxicity in our experimental model (Supplementry Figure S3). Therefore, we decided to use a genetic approach based on shRNA. As shown in Figure 5B and 5C, we achieved an effective knock down at the RNA and protein levels for MKP1 in E1a expressing cells, which again correlated with basal activation of p38MAPK and ERK1/2 signaling pathways, (Figure 5D) and restored resistance similar to control cells (Figure 5E).

Finally, to fully clarify the role of MAPKs in the chemosensitizer effect of E1a we took advantage of the specific inhibitors for both MAPK signaling pathway affected by E1a, ERK1/2 and p38MAPK, such as PD98059 and SB203580 [36, 37]. To this end, E1a expressing and non-expressing cells were incubated in the presence or absence of MAPK inhibitors with increased doses of cDDP. As shown (Figure 5F), the ERK1/2 signaling pathway inhibitor did not modify significantly the response to cDDP, regardless the presence or absence of E1a. However, SB203580 (Figure 5G) promoted an increase in the sensitivity of E.V. that was not observed in E1a positive cells.

Taken together, these data indicate that, in E1a NSCLC expressing cells, upregulation of MKP1 and the subsequent p38MAPK inhibition is required for the induction of chemosensitivity to cDDP. In addition, the present data also suggest that ERK1/2 signaling pathway is not directly implicated in the chemosensitizer effect associated to E1a

## DISCUSSION

Several conclusions can be drawn from the present report.

First, E1a is able to promote sensitivity to cDDP in different models of NSCLC. This observation increases the list of tumor models in which E1a is able to induce chemosensitivity to cDDP as in the case of Squamous Cell Carcinoma or Ovarian cancer. [6, 38] Furthermore, this observation reinforces the universal character of E1a as therapeutic agent regardless of the tumor type. In addition, our observations indicate how E1a is able to promote chemosensitivity in different experimental models with different genetic patterns [30]. In this sense the tumor suppressor p53 has been considered as a master gene in the cellular response to cDDP since almost 20 years ago [39] with clinical implications as a bio marker for resistance to cDDP in lung cancer as well as in other pathologies [40–42]. Our data in H1299 cells, with a genetic lack of p53 gene, support the use of E1a as chemosensitizer agent in the presence of non-functional p53, which could account up to for 50% of human tumors. Even more, other genetic hallmarks of lung cancer such as mutant K-Ras [43], known to been implicated in cDDP resistance [44], seem to be not implicated in the effect exerted by E1a, as the data in H23 cell line, with a mutant K-Ras gene [45], indicates. Therefore, our data supports the use of E1a as new universal chemosensitizer agent in lung cancer, especially in those cases with alterations in key genes that show an acute resistance. In addition, we consider that the evaluation of MKP1 expression, in agreement with previous reports that support the use of MKP1 as a biomarker in different types of tumors, [29, 46] could be key point in a future therapy based on E1a.

Regarding to MKP1 our data support how this phosphatase is a key player in the cellular response to cDDP and how cells with low levels of MKP1 have an intrinsic resistance to cDDP. In this sense, previous evidence supported MKP1 down-modulation as a mechanism to induce drug sensitivity in NSCLC [34, 47–49]. However in our experimental model of NSCLC we observed that low endogenous levels of MKP1 correlates with a marked “*de novo*” resistance. In agreement with this observation, E1a increases the level of MKP1 to promote sensitivity. In addition is noteworthy that E1a is not only affecting MKP1 dependent signaling, other pathways, as the PI3K/AKT pathway [10], could be modulated by E1a to account for the chemosensitive phenotype observed. In this regard, considering that MAPKs are the natural substrates of MKP1, our data support a role for p38MAPK in the chemosensitizer effect of E1a. Indeed, the p38MAPK signaling pathway, also known to be a target of E1a, is related to cDDP response [11, 19] and is noteworthy that recent evidence supports that the inhibition of p38MAPK is able to promote sensitivity to cDDP [50], in agreement with our observation in the context of E1a expression. However our data do not support a direct implication of the ERK1/2 in the chemosensitizer effect of E1a, in spite of being a signaling pathway targeted by E1a [20, 51] and related to cDDP resistance [52, 53]. Nonetheless, other mechanisms should be considered to fully explain the role of MKP1 in the cellular response to cDDP in the presence of E1a. For example, it is well stablished that NF-kb inhibition mediates chemosensitivity [54, 55] and its activation correlates with resistance [56]. Interestingly, NF-kb pathway is also targeted by E1a [57] and recent evidences showed an inverse correlation between MKP1 expression and NF-kb activity [58] which could account for our observations in E1a associated sensitivity. Furthermore, possibilities as deregulation in proteins like RIP [59] could account for the chemosensitizer effect of E1a if mediated trough NF-kb, especially considering the relationship between E1a and the JNK signaling pathway [21]. Therefore, further studies are required to fully elucidate the mechanism affected by E1a to promote cDDP sensitivity. In addition, is interesting to mention how the upregulation of MKP1 has been considered as a critical step in novel therapeutic approaches for lung cancer, as in the case of K-Ras driven lung carcinogenesis and γ-secretase [60].

Finally is important to mention that E1a is able to promote cDDP sensitivity trough an increase in apoptosis, that correlates with a blockage of the autophagic response associated to cDDP resistance [61, 62]. This issue raises the possibility that E1a could be a novel negative modulator of autophagy associated to cDDP by promoting apoptosis. However, it has been proposed that E1a plus E1b are required for adenovirus associated autophagy [63]. In this sense our data show how cells expressing only E1a showed an apparent increase in the basal flux of autophagy, by means of higher level of LC3 lipidated and low p62 levels as well as the lack of response to chloroquine. Therefore, this deregulated basal autophagic flux could explain the observed lack of an apparent autophagic response in case of H1299 E1a expressing cells. Nonetheless this hypothesis, as well as the implication of MKP1 in a putative deregulation in the basal autophagy flux, needs to be further investigate to fully stablish the role of E1a in autophagy.

In summary, here we present evidences showing how MKP1 upregulation is a critical event in E1a associated sensitivity to cDDP in a NSCLC experimental model. This effect is independent of key genes in lung carcinogenesis as p53 or K-Ras and is mediated with an increase in the cDDP triggered apoptosis. Therefore, our data support the use of E1a gene in future therapeutic approaches for lung cancer, specifically in cDDP resistant tumors. Whether our observation could be extrapolated to other type of tumors or therapeutic agents need to be deeply investigated.

## MATERIALS AND METHODS

### Cell lines and plasmids

Non-small lung cancer cells (H23, H460, H661 and H1299) were maintained in DMEM supplemented with 10% FBS plus antibiotics (Sigma-Aldrich, Tres Cantos, Madrid, Spain). Cells were maintained in 5% CO_2_ and 37°C. H460 and H1299 cell lines were purchased from ATCC (LGC Promochem, Barcelona, Spain). H23 and H661 cell lines were kindly provide by Dr. R. Pio (Department of Biochemistry, School of Sciences. University of Navarra, Pamplona, Spain). All these cell lines have been previously described [64].

To generate H460 resistant cell line by co-culturing with cDDP, cells were routinely maintained with different concentration of cDDP (ranging from 0.1 μg/mL to 0.8 μg/mL) during 2 months. After that, the H460 cells that were able to survive in the highest concentration of cDDP (0.2 μg/mL) were used for further experiments.

E1a isoform 13s was obtained by PCR from cDNA of 293T cells and cloned in PCDNA3.1 and then subcloned into pLESIP vector. This construct has been previously described [9]. pLESIP-E1a 12s was obtained by PCR cloning from pLPC vector kindly provided by Dr. Scott Lowe's lab (Memorial Sloan-Kettering Cancer Center, New York, New York USA). PCR Primers used were; forward 5′- GTGGATCCATGAGACATATTATCTGCC-3′ and reverse 5′-GGGAATTCTTATGGCCTGGGGCGTTTAC-3′. PCR conditions were 94°C 30 sec. during the first cycle and then, 30 cycles (94°C 30 sec, 52°C 1 min and 72°C 3 min) with a final extension of 72°C during 10 min. After sequencing and expression assay, E1a 12s was cloned, using the BamH1/EcoR1 sites, onto pLESIP vector.

### Chemicals and antibodies

Antibodies against active form of p38 as well as total p38 were from Cell Signaling Technologies (Izasa, Barcelona, Spain). Antibodies against E1a, p62, MKP1 and tubulin were purchased from Santa Cruz Technology (Quimigen, Madrid, Spain). Antibody against LC3 was from Sigma Aldrich (Tres Cantos, Madrid, Sapin). cDDP was purchased from Ferre Farma (Barcelona, Spain) and prepared freshly before use. The inhibitors for MKP1, (Ro 31–8220 Mesylate), p38MAPK (SB 203580) and ERK1/2 (PD098059) were purchased from Selleckchem (DeltaClon, Madrid, Spain), dissolved in DMSO at 10 mM, aliquoted and stored at −20°C.

### Transfections and infections

Lentiviral production and infection was performed as previously described [9, 64]. Briefly, HEK293T cells were transfected overnight using calcium phosphate with 9 μg of pLESIP E1a 13s, pLKO-shRNA for MKP1 or pLKO empty vector plus 6μg of PSPAX2, and 3 μg of the viral envelope protein (VSVG). Supernatant was collected 48 hours after transfection. Host cells were infected by adding packaging cells media in the presence of 4 μg/ml polybrene from Sigma-Aldrich. 48 hours after infection cells were exposed to 2 μg/ml of puromycin (Sigma-Aldrich) for H460, 1 μg/ml for H23, 1 μg/ml for H661 and 3 μg/ml for H1299 cells, for at least 3 days before any assay. Infected cells were routinely maintained at the appropriate concentrations of puromycin.

Transfection of pCDNA3 E1a 13 was performed with 2 μg of the plasmid by using lipofectamine 2000 (Invitrogen, Barcelona Spian). After 48 h, cells were selected with G418 (Sigma-Aldrich) at 800 μg/ml, for at least 10 days, prior any assay.

### Western blotting

Cells were collected in lysis buffer (100 mM HEPES, pH 7.5, 50 mM NaCl, 0,1% Triton X-100, 5 mM EDTA, 0.125 M EGTA). Protease and phosphatase inhibitors (0.2 μg/ml Leupeptin, 2 μg/ml, Aprotinin, 1 mM PMSF and 0.1 mM Na_3_VO_4_) were added prior to lysis. Protein quantification was performed by using the BCA Protein Assay Kit (Pierce, Madrid, Spain) following the manufacturer's instructions. Indicated amounts of protein were loaded onto 10% SDS-PAGE, transferred to PVDF membranes and blotted against different proteins using specific antibodies.

Antibody detection was achieved by enhanced chemiluminescence (Amersham, GE Health Care, Barcelona, Spain) in a LAS-3000 system (FujiFilm, Japan). Results show a representative blot out of three with nearly identical results. Tubulin was used as a loading control. Images show a representative experiment out of 3, with nearly identical results. Quantification was performed by using NIH ImageJ software.

### RNA isolation, reverse transcription and quantitative real-time PCR

Total RNA was obtained and reverse transcription was performed as previously described [65]. Changes in the mRNA expression of MKP1 and DUSP5 were examined by Quantitative Real-Time PCR using an ABIPrism 7500 FAST Sequence Detection System (Applied Biosystems, Madrid, Spain). cDNA was amplified using SYBR Green I PCR Master Mix (Applied Biosystems) in the presence of specific oligonucleotides. The PCR conditions and quantification was performed as previously described. [65] Primers for all target sequences were designed using the computer Primer Express software specially provided with the 7000 Sequence Detection System (Applied Biosystems). PCR primers were purchase from Bonsai Technologies (Madrid, Spain).

For MKP1: forward 5′-AGCCACCATCTGCCTT GCTTA-3′; reverse ′5′-CTGGCCCATGAAGCTGAAG TT-3′.

For DUSP5: forward 5′-CGGAATATCCTGAGTG TTGCG-3′ - reverse 5′-CACTTGGATGCATGGTAGG CA-3′ and for GAPDH: forward 5′-TCGTGGAAGGACTC ATGACCA-3′, reverse 5′-CAGTCTTCTGGGTGGCAG TGA-3′. Data are the average of, at least, three independent experiments performed in triplicate.

### Interference assays

Plasmids containing the sh-RNA for MKP1 were purchased from Sigma-Aldrich (SHCLND-NM_004417) and used following the manufacturer's recommendations as previously described [62]. Five different clones were tested and best performing clone was selected for further experiments.

### Viability assays

Viability was evaluated by crystal violet method and confirmed by MTT assay [62]. For crystal violet method, cells were seeded 24 hours prior drug treatment at 2 × 10^4^ cells/well. Colorant was recovered using 1% acetic acid and optical density was evaluated at 590 nm. Values were referred to untreated controls. Data are the average of at least 3 independent experiments performed in triplicate cultures.

The MTT assay were performed with a 2 × 10^4^ cells/well plated in 24-well plates and then exposed to tested agents. MTT reactant (Thiazolyl Blue Tetrazolium Bromide, M2128, Sigma Aldrich), at 5 mg/ml in a PBS solution, was added to the cells in a 1:10 ratio (MTT solution:culture medium) and left for incubation during 1 h at 37°C. After removal of the medium, DMSO was added to each well to dissolve the formazan crystals. The absorbance at 540 nm was determined using a Biokinetics plate reader (Bio-Tek Instruments, Inc, Winooski, VT, USA). Data are the average of at least 3 independent experiments performed in triplicates cultures.

### Apoptosis assays

For caspase 3/7 assays, cells were plated at a density of 10^4^ cells/well in opaque 96-well plates 24 hours prior to treatment. 24 hours after treatment, activation of effector caspases 3 and 7 was evaluated with Promega's CaspaseGlo kit (G8090) as previously described [62]. Resulting mixtures were quantified after 30 minutes of incubation at room temperature in a Beckton Dickinson BD 3096 luminometer. Data are the average of at least 3 independent experiments performed in triplicates cultures.

### Statistical analysis

Data are presented as mean ± S.D. Statistical significance was evaluated by Student's *t* test using the GraphPad Prism 5.00 software. The statistical significance of differences was indicated in figures by asterisks as follows: *P < 0.05, **P < 0.01 and ***P < 0.001.

## SUPPLEMENTARY MATERIAL FIGURES



## Abbreviations

cDDP: Cisplatin
NSCLC: Non-small cell lung carcinoma
MAPK: Mitogen Activated Protein Kinase
DMSO: Dimethyl sulfoxide
SCC: Squamous cell carcinoma
VSVG: Viral envelope protein
TCL: total cell lysates
E.V.: empty vector
