# Supplementary material for: MKP1 mediates chemosensitizer effects of E1a in response to cisplatin in non-small cell lung carcinoma cells
Source: Oncotarget. 2015 Dec 12;6(42):44095–107. doi: 10.18632/oncotarget.6574 (PMC4792544; doi:10.18632/oncotarget.6574)
Supplement: Supplementary file 1 [file oncotarget-06-44095-s001.pdf]

MKP1 mediates chemosensitizer effects of E1a in response to cisplatin in non-small cell lung carcinoma cells

Supplementary Materials

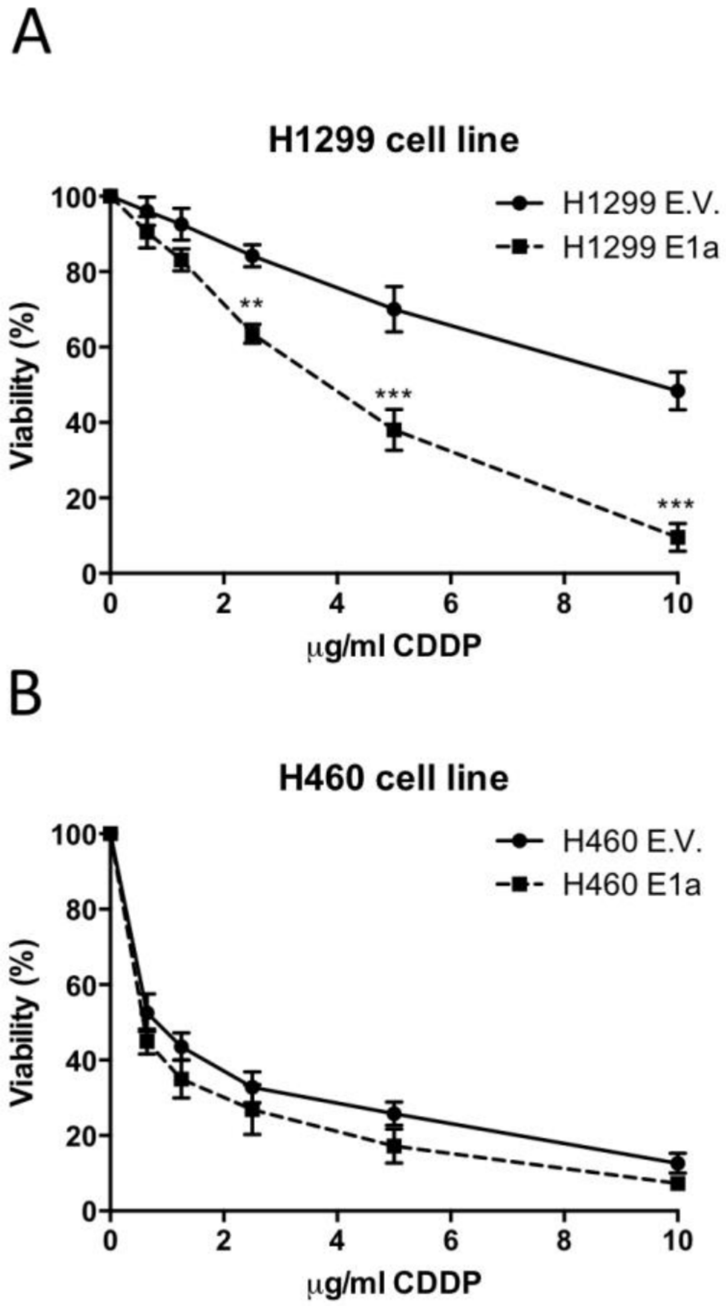

Supplementary Figure S1: Viability assay (MTT based assay) in H1299 and H460 E.V. and E1a 13 s cells in response to cDDP at indicated doses for 48 h. Viability was referred to untreated cells as 100%. Data represent the average ± SD.

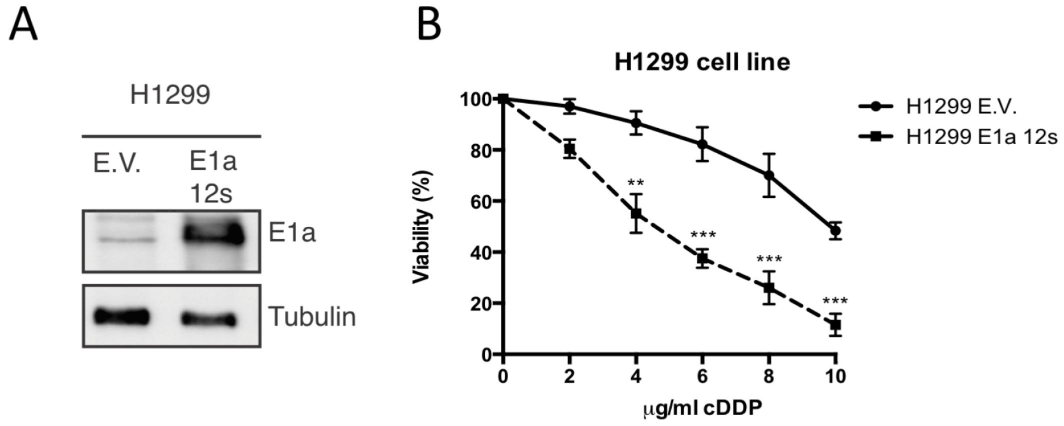

Supplementary Figure S2: H1229 cells were infected with lentivirus carrying the 12 s isoform. Expression was evaluated by western blotting (**A**) and viability assay (MTT based assay) in response to cDDP (**B**) was performed at indicated doses for 48 h. Viability was referred to untreated cells as 100%. Data represent the average ± SD.

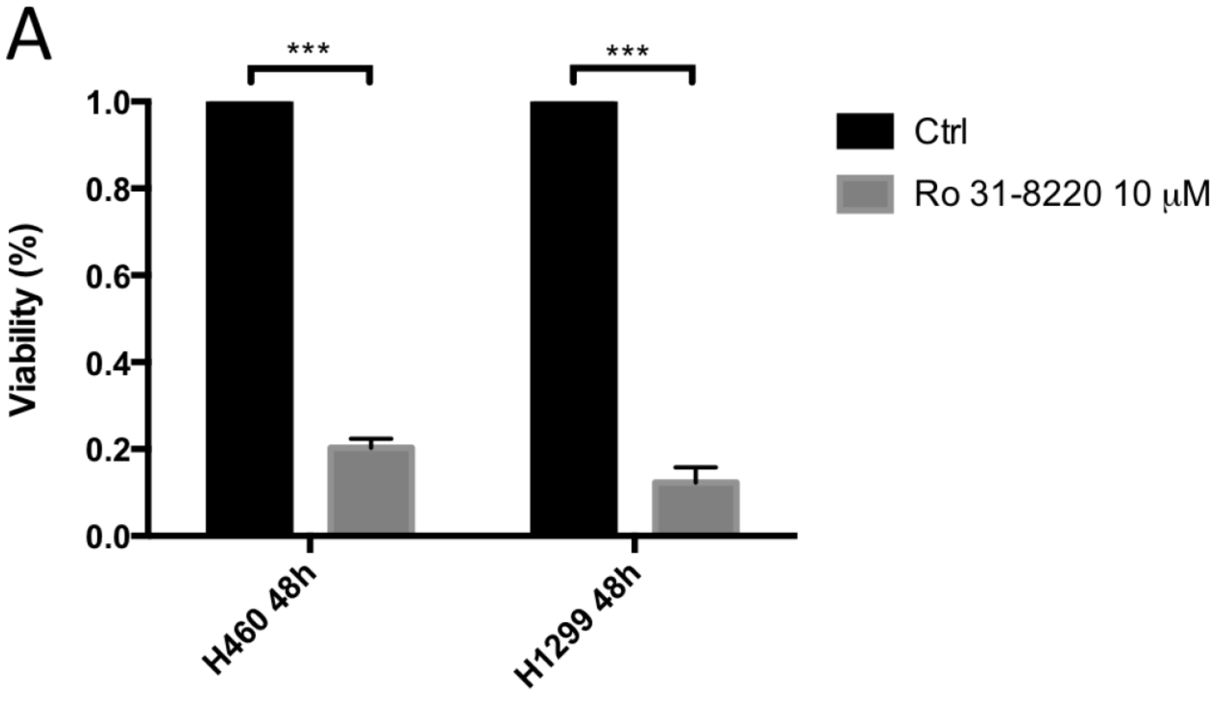

Supplementary Figure S3: H1299 and H460 were treated with Ro-31-8220 for 48 hours and then viability was evaluated by crystal violet method.
